# BOiS—Berlin Object in Scene Database: Controlled Photographic Images for Visual Search Experiments with Quantified Contextual Priors

**DOI:** 10.3389/fpsyg.2016.00749

**Published:** 2016-05-23

**Authors:** Johannes Mohr, Julia Seyfarth, Andreas Lueschow, Joachim E. Weber, Felix A. Wichmann, Klaus Obermayer

**Affiliations:** ^1^Neural Information Processing Group, Department of Electrical Engineering and Computer Science, Technische Universität BerlinBerlin, Germany; ^2^Cognitive Neurophysiology Group, Department of Neurology, Campus Benjamin Franklin, Charité-University Medicine BerlinBerlin, Germany; ^3^Neural Information Processing Group, Faculty of Science, Bernstein Center for Computational Neuroscience Tübingen, University of TübingenTübingen, Germany; ^4^Empirical Inference Department, Max Planck Institute for Intelligent SystemsTübingen, Germany; ^5^Bernstein Center for Computational Neuroscience BerlinBerlin, Germany

**Keywords:** photo database, visual search, contextual prior, realistic scenes, target object

## Introduction

Photographic stimuli are often used for studying human perception. To faithfully represent our natural viewing environment, these stimuli should be free of potential artifacts. If stimulus material for scientific experiments is generated from photographs that were created for a different purpose, such as advertisement or art, the scene layout and focal depth might not be typical for our visual world. For instance in advertising photos, particular objects are often centered and focused. In visual search experiments, this can lead to the so-called central viewing bias and an unwanted pre-segmentation of focused objects (Wichmann et al., 2010). Also the photographic process itself can result in artifacts, such as optical, color and geometric distortions, or introduce noise. Furthermore, some image compression methods introduce artifacts that may influence human viewing behavior. In some studies, objects are pasted into scenes using graphics editing. In this case inconsistencies in color, shading or lighting between the object and the local scene background could lead to deviations from natural viewing behavior.

In order to meet the needs for publicly available stimulus material in which these artifacts are avoided, we introduce in this paper the BOiS—Berlin Object in Scene database, which provides controlled photographic stimulus material for the assessment of human visual search behavior under natural conditions. The BOiS database comprises high-resolution photographs of 130 cluttered scenes. In each scene, one particular object was chosen as search target. The scene was then photographed three times: with the target object at an expected location, at an unexpected location, or absent.

Moreover, the database contains 240 different views of each target object in front of a black background. These images provide different visual cues of the target before the search is initiated. All photos were taken under controlled conditions with respect to photographic parameters and layout and were corrected for optical distortions.

The BOiS database allows investigating the top-down influence of scene context, by providing contextual prior maps of each scene that quantify people's expectations to find the target object at a particular location. These maps were obtained by averaging the individual expectations of 10 subjects and can be used to model context effects on the search process.

Last not least, the database includes segmentation masks of each target object in the two corresponding scene images, as well as a list of semantic information on the target object, the scene, and the two chosen locations. Moreover, we provide bottom-up saliency measures and contextual prior values at the two target object locations. While originally aimed at visual search, our database can also provide stimuli for experiments on scene viewing and object recognition, or serve as test environment for computer vision algorithms.

## Photographic images

### Target objects in realistic scenes

**Figure 1:**
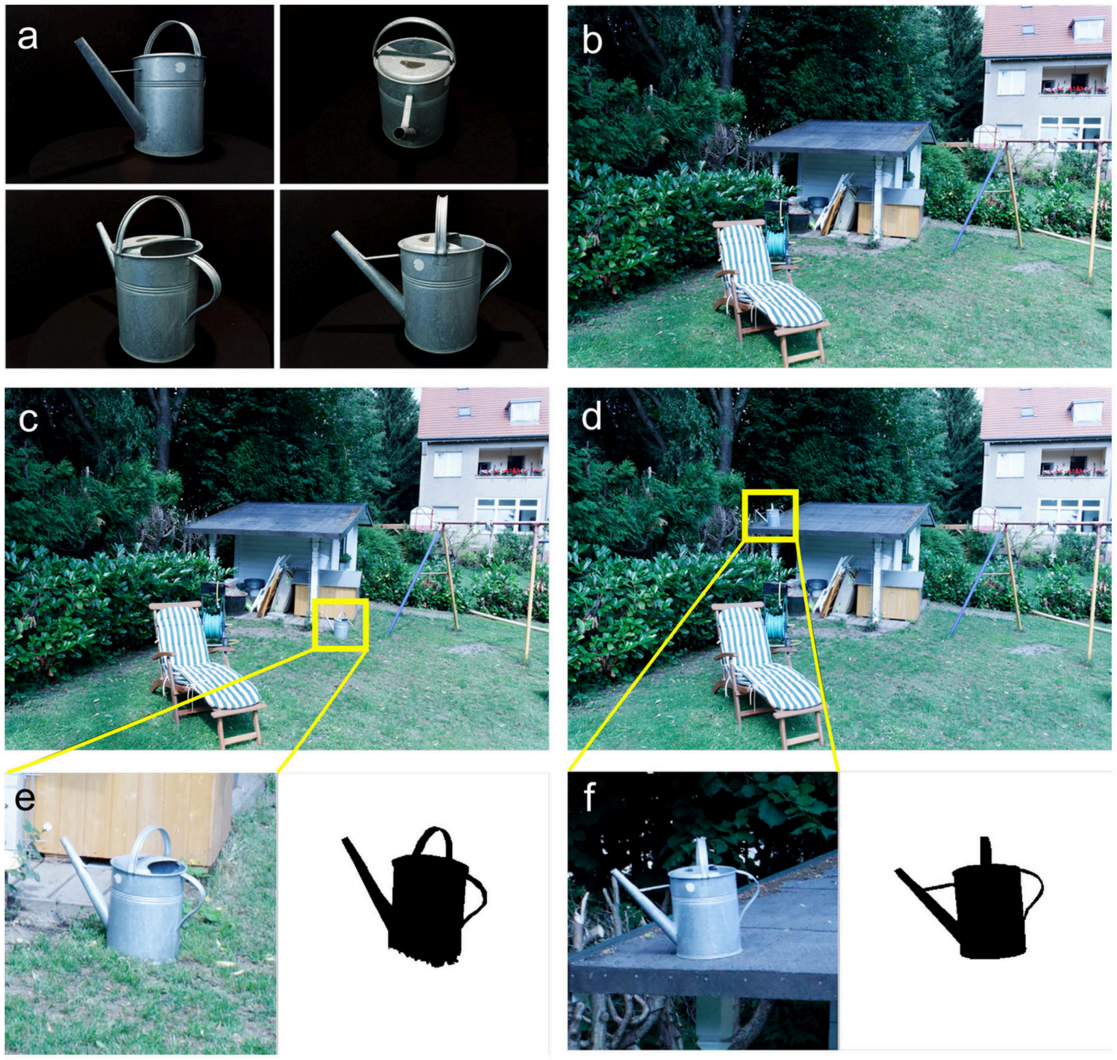
**Examples of target object in scenes: watering can in the garden. (A)** Example of multiple views of the target object (watering can). All target objects were characterized by 80 views under rotation around the vertical axis and from 3 vertical viewing angles. Each scene is photographed in three versions: target object is **(B)** missing, **(C)** in an expected location and **(D)** in an unexpected location. In these images we have marked the locations of the target objects by boxes, which are not present in the original images. The bottom row **(E,F)** shows a magnification of these boxes and the segmentation of the target object in the particular location.

Our goal was to obtain scenes that were as naturalistic as possible, using visual environments encountered in everyday life. Therefore, for each of the 130 scenes a particular target object was chosen from among the objects contained within the scene, such as a pen lying on a desk, a sofa cushion in a living room or a watering can in a garden (see the example in Figures 1A–D). About 75% of the scenes were exactly photographed as found, while 25% of the scenes were partly manually arranged on purpose, providing different target object locations and making the search for the target sufficiently challenging. In this case, objects found in the environment were rearranged, e.g., a cupboard door was opened to make a target object inside of the cupboard visible, or a tidy desk was disarranged to increase the clutter level of the scene. The 130 target objects were all semantically associated to the scene they appear in. Each scene was photographed in three versions: The first version of the scene (Figure 1B) did not contain the target object (Figure 1A) at all. In the second version (Figure 1C), the target object was placed at a location within the scene where people might expect to see it. In the third version (Figure 1D), the target object was located at a less likely location. The three versions of a scene were photographed from exactly the same viewpoint using a tripod to ensure that they were essentially identical except for the target object. The two target object locations were chosen based on our subjective judgment; however people's expectations about target object location within the scene were quantified *post-hoc* by contextual prior maps (see Section Contextual Prior Maps). For each of the 130 target objects the BOiS-database contains three versions of one particular scene, resulting in a total number of 390 scene images.

Each scene in our database is labeled as indoor- or outdoor-scene and assigned to a further sub-category. The 107 indoor scenes that are subdivided into 32 kitchen scenes, 12 bathroom scenes, 9 bedroom scenes, 16 living room scenes, 15 office scenes, 8 hall scenes, 7 hospital scenes, 5 garage scenes, 2 bakery scenes, and 1 shop scene. The 23 outdoor scenes are subdivided into 13 garden scenes, 4 park scenes, 4 terrace scenes, 1 garage scene, and 1 street scene. For each scene, we also name the associated target object, and describe the two locations where the target object was photographed within the scene by their relation to nearby objects. For the “expected” location there usually were some semantically associated objects next or close to the target object. For instance, the bottle cap was screwed onto a bottle, the rubber duck was sitting on the rim of a bathtub, or the garden watering can was standing next to a garden shed. For the “unexpected” location, the surrounding objects did not have a semantic relation to the target object. For example, the bottle cap was lying on top of washed dishes, the rubber duck was sitting on the bathroom floor, and the garden watering can was standing on top of the garden shed.

In order to avoid potential artifacts, no graphics editing was applied and target objects were physically placed at different locations within the scene. No humans or animals appeared in the pictures in order to avoid any attentional biases caused by their presence (Suzuki and Cavanagh, 1995).

### Layout of scenes

All scenes were taken from a natural human perspective. The layout of all scenes was similar: The central area and the outer margin of the image were kept clear of the target object. This allows for generating different cuts of the original scene by zooming and shifting, providing more control over size and relative position of the target object in the final stimulus. Furthermore, it was ensured that the two locations for one particular target object had a similar distance to the image center (minimum distance at expected location: 605.6 ± 88.1 pixels, at unexpected location: 618.1 ± 93.5 pixels; no significant difference, *p* = 0.27). We ensured that the expected and unexpected target object locations have a similar spatial distribution over all scenes. Thus, we avoided a bias such as all expected locations being in the lower half and all unexpected locations being in the upper half of the image. All target objects fit in a square bounding box whose side length ranged from about 1/30 to 1/10 of the image height. In order to obtain a similar size for one particular target object at both object locations within one scene we tried to place the camera at a roughly similar distance to both locations. If parts of a target object were occluded in one image, we tried to occlude similar parts in the corresponding image with the target object at the other location.

### Separate images of target objects

Target objects had a varying absolute size, ranging from a few millimeters up to about 60 cm, such as a coin, a pair of scissors, a cooking pot, or a car tire. We acquired multiple views of each target object in front of a black background (see Figure 1A for an example). The target objects were placed individually on table rotating in steps of 4.5° once around the vertical axis. This was done for three different vertical viewing angles of the camera - 0°, 22.5°, and 45°. Therefore, each target object was described by 240 views. Flat objects were photographed only under vertical viewing angles of 22.5° and/or 45°, and some objects were also photographed upside down. In total, our database contains 30,160 object images. The large number of different views allows the selection of particular views of a target object as cue in visual search experiments. One could even cue by an animated rotation of the target object.

### Image acquisition

For all images we used the same hardware and pre-defined setting ranges. All photographic images of scenes and target objects were taken with a Canon EOS 450D camera with a Canon EF-S 18-55/3.5-5.6 IS lens. Shutter priority was fixed at F 9.5, exposure time ranged from 5 to 1/1500 s, and ISO speed rating was either ISO 400 or ISO 800, depending on lighting conditions. Photoflash was only used when indoor lighting conditions were inadequate. Focal length was 18 mm; in some images zooming was used with a focal length of up to 33 mm. All pictures were recorded at a high resolution of 4272 × 2848 and saved in Canon RAW image file format (.CR2). Taking the particular lens into account, all photos were automatically corrected for optical errors (optical and geometric distortion, vignetting, chromatic aberrations, lens softness, noise reduction, white balancing) using DxO Optics Pro 9.

## Contextual prior maps

One important top-down influence arises from scene context (Castelhano and Heaven, 2011). Local scene context helps us in determining where in the scene the particular target object is likely to appear based on the relation to the other objects in the scene. In order to allow investigating the influence of local scene context on visual search, contextual prior maps of each scene were included in the BOiS-database. These were obtained by quantitatively capturing the common expectations people have about the location of a target object within a given scene. One application of these contextual prior maps is to differentiate top-down local scene context effects from bottom-up saliency effects in experiments of visual search.

**Figure 2:**
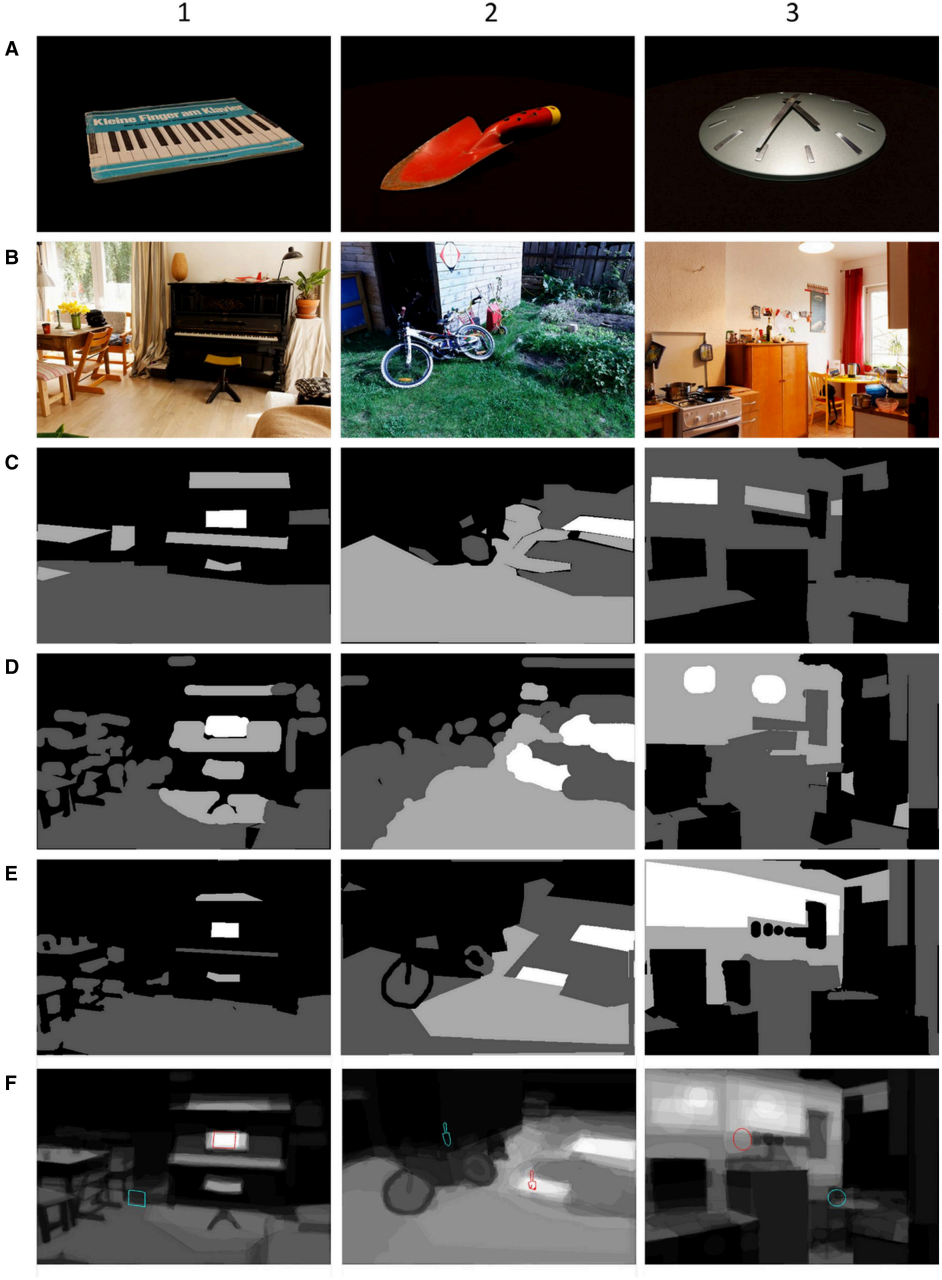
**Contextual prior maps for three different target objects and corresponding scenes: music book for piano (1), shovel (2), clock (3)**. The rows show the target object **(A)**, the scene where the target object is absent **(B)**, **three** examples of individual contextual prior maps **(C–E)** and the average contextual prior maps of 10 subjects **(F)** with an overlay of the segmentation outlines of the target object (the red line marks the expected location and the blue line marks the unexpected location). The maps are encoded as gray scale images (white for most likely, black for most unlikely).

For this purpose, 10 naïve subjects (5 male and 5 female students living in Berlin, between 20 and 30 years old) segmented the scenes with missing target objects (Figures 2A,B) into regions of different likelihood for containing the target object. The images were segmented into regions using different colors representing four different levels of likelihood (most likely, likely, unlikely, most unlikely). Segmentations were done as pixel labeling using a set of graphics editing tools: magnifying glass, brush, lasso, and a polygon selection tool. Subjects were instructed to take the physical extension of the target object into account and mark the pixels where the target object might be visible. An individual contextual prior map was obtained for each subject and scene by mapping all pixels to values 0 (most unlikely), 1/3 (unlikely), 2/3 (likely), and 1 (most likely; Figures 2C–E). Finally, for each of the 130 scenes a contextual prior map was calculated by averaging the individual maps of all subjects (Figure 2F). In our database the contextual prior maps are provided as grayscale images (white for most likely, black for most unlikely) that need to be normalized to sum to 1 to make the map values comparable across the scenes. Then each pixel value reflects the relative amount of contextual prior that can be attributed to that particular pixel.

## Target object segmentation in scenes

We provide segmentations of the target objects in the scenes and use these to include values for several bottom-up features that are potentially related to the detectability of the target object within the scene.

Each target object is associated with a particular scene, and appears in two images of this scene. Our database contains binary masks for these scene images in which the pixels belonging to the target object are marked. These masks were obtained by exact manual segmentation in the high resolution image and are therefore very accurate (for an example see Figures 1E,F). One can use these masks to measure features or calculate image statistics within the area of the target object. In eye tracking studies, one can use these masks to check whether the target object was targeted by a saccade. In addition, the segmentations provide a ground truth for computer vision algorithms.

We applied these target object segmentation to the contextual prior maps in order to validate our subjective assessment of expected and unexpected target object location. For each of the 130 pairs, we averaged the contextual prior for both the expected and the unexpected target object location over the pixels taken up by the object. Thus, each version of the scene was represented as a single number reflecting how expected the target object was for that particular location. The difference in this value across the different version of the scene will tell us about the differences in expectation. A paired *t*-test showed a significant difference between the expected and the unexpected target object locations (*p* < 10^−38^). This was also the case for the maximum value of the contextual prior over the pixels taken up by the object (*p* < 10^−38^).

In 129 of the scenes the expected target object locations had a higher maximum contextual prior value than the corresponding unexpected target object location. For these scenes, our subjective assessment was thus confirmed. In one scene our categorization into “expected” and “unexpected” location did not correspond to the results of contextual prior maps (scene 118). When using our database to study prior information on visual search it may thus be prudent to exclude scene ID 118.

## Bottom-up features

Our database includes a datasheet listing a set of values for bottom-up features that are potentially related to the detectability of target object within a scene: target object size; minimum and mean distance to image center; conspicuity map values for luminance, color and orientation; saliency of the target object.

Larger size of an object increases its visibility within a scene and its likelihood of coincidental detection. Therefore, the object size could modulate attention. We defined the size of the target object as the number of pixels covered by the target object mask.

When looking at images humans tend to first fixate the area close to the center of the image (central fixation bias). Therefore, the distance to the image center might be inversely correlated to the detectability of the target (Tatler and Melcher, 2007). We measured the minimum and mean distance of all pixels within the object mask to the image center.

Saliency maps form the basis of standard models of bottom-up visual attention. We calculated conspicuity maps and saliency map based on the model of Itti and Koch (Itti et al., 1998) using the Matlab Saliency Toolbox (Walther and Koch, 2006) after resampling images to a resolution of 1200 × 800 pixels. The algorithm first extracts feature maps for color, intensity and orientation channels by calculating center-surround differences between different levels of Gaussian pyramids and normalizing. These are then combined across scales and normalized to yield conspicuity maps. The final saliency map is then formed by a linear combination of the three channels. We normalized the values of the conspicuity maps and the final saliency map such that the sum over all pixels had the value 1. Using this normalization, pixel values reflect the relative amount of attention that according to the saliency model is focused on a particular pixel. For analyzing and comparison final saliency and conspicuity maps need to be resampled to the size of original pictures. We calculated the mean, median and maximum values of the individual conspicuity maps for intensity, color and orientation, and the final saliency map within the segmentation mask of the target object.

## Database details

The database is publicly available under the following address: http://info.ni.tu-berlin.de/photodb/. It can be downloaded as a single archive bois_db.zip (~24 GB). Alternatively, all files belonging to the particular target object ids can be downloaded as separate archives (TO_ < id>.zip) by clicking on the corresponding id in a table on the website. Both the single archive as the individual archives unpack into the same directory tree. The root directory is called “bois_db” and has four subfolders.

The first folder “original_scenes_DxO” contains the distortion corrected scene images as PNG files with a resolution of 4272 × 2848 pixels. The scenes without target object are named O_ < id>L_DxO.png, the scenes with the target object at the expected location O_ < id>E_DxO.png, and the scene with the target object at the unexpected location O_ < id>U_DxO.png. The second folder “target_objects” contains a subfolder DxO, in which for each target object < id> one can find a folder TO_ < id>_DxO with the corrected PNG images of the different views of the target objects, TO_ < id>_ < vertical angle>_ < horizontal viewpoint>_DxO.png. These were down-sampled to 1152 × 786 pixel resolution in order to keep the total size of the database practicable. The third folder “masks” contains the segmentation masks of the scenes with the target object in expected (O_ < id>E_m.png) and unexpected (O_ < id>U_m.png) location at 4272 × 2848 pixel resolution. The last folder “cpms” contains the contextual prior maps as gray level images (CPM_ < id>.png) at 4272 × 2848 pixels resolution. Note that these maps are scaled to use the whole gray value range for visualization purposes and need to be normalized such that the sum over all pixels is 1 to ensure comparability across scenes. We have also included the 130 individual contextual prior segmentations of our 10 subjects in our database at 600 × 400 pixels resolution (Individual_priors.zip). These files are named < subject_id>_O_ < id>L_s.png, where < subject_id> ranges from 2 to 11, and the gray values (0, 85, 170, 255) in the images encode the four prior probability levels from lowest to highest.

All meta-information for the database is provided in BOiSmeta.xls. This Excel spreadsheet contains a table with the semantic information (see Section Target Objects in Realistic Scenes), as well as a table with the contextual prior values (see Section Contextual Prior Maps) and bottom-up feature values (see Section Bottom-Up Features) for both target object locations within the scene.

## Author contributions

All authors jointly developed the concept of the database. JS generated all the photos. JS and JM compiled and annotated the database, and wrote the manuscript. AL, JW, FW, and KO carefully revised the manuscript for important intellectual content.

## Funding

This work was funded by the German Federal Ministry of Education and Research (BMBF) through the Bernstein Focus: Neurotechnology (FKZ: 01GQ0850 – A4) as well as the Bernstein Computational Neuroscience Program Tübingen (FKZ: 01GQ1002).

### Conflict of interest statement

The authors declare that the research was conducted in the absence of any commercial or financial relationships that could be construed as a potential conflict of interest.
